# Cognitive behavioral group therapy for patients with physical diseases and comorbid depressive or adjustment disorders on a waiting list for individual therapy: results from a randomized controlled trial

**DOI:** 10.1186/s12888-017-1494-9

**Published:** 2017-10-10

**Authors:** Miriam Ruesch, Almut Helmes, Juergen Bengel

**Affiliations:** Department of Rehabilitation Psychology and Psychotherapy, Institute of Psychology, Albert-Ludwigs-University Freiburg, Engelbergerstr. 41, 79085 Freiburg, Germany

**Keywords:** Waiting lists, Cognitive behavioral group therapy, Physical diseases, Depression, Adjustment disorders

## Abstract

**Background:**

Depressive and adjustment disorders are highly prevalent in patients with physical diseases and are associated with poorer quality of life, increased morbidity and mortality, as well as higher healthcare costs. Access to mental health care holds strong importance for these patients, although waiting times for outpatient individual psychotherapy in Germany are often long. Attending an intervention while waiting for individual therapy could improve this problem. For this purpose, we developed an eight-session cognitive behavioral group therapy (STEpS) and tested its efficacy in a randomized controlled trial.

**Methods:**

Seventy-six patients with chronic physical diseases and comorbid depressive or adjustment disorders were randomized to either STEpS or a waiting list control group. The primary outcome was self-reported depression measured by the Hospital Anxiety and Depression Scale (HADS-D), while the secondary outcomes included global psychological distress and health-related quality of life. Data was assessed at baseline, post-treatment and 2-month follow-up and was analyzed based on intention-to-treat.

**Results:**

Compared to the control group, the STEpS group showed significantly less depression (*d* = 0.37; *p* = .009) and significantly higher quality of life (mental: *d* = 0.47; *p* = .030; physical: *d* = 0.70; *p* = .001) at post-treatment. The groups did not differ in global psychological distress. At 2-month follow-up, the STEpS group indicated significantly higher subjective physical health (*d* = 0.43; *p* = .046), but did not differ from the control group in the remaining outcomes.

**Conclusions:**

STEpS proved effective in improving depression and health-related quality of life in the short term but did not reveal effects on mental outcomes at 2-month follow-up. Nonetheless, the implementation of STEpS as a waiting list intervention prior to individual therapy could help patients to handle long waiting periods in outpatient care.

**Trial registration:**

German Clinical Trials Register DRKS00005140 (27 August 2013).

## Background

Imagine that you are diagnosed with a chronic disease and realize at some point that it is too difficult to cope on your own. You contact a psychotherapist and he tells you that you have to wait 6 months until you can begin your therapy. You would probably feel rather left alone. This is what happens to many patients in Germany. According to a survey based on data of 8620 outpatient psychotherapists, waiting times in Germany are on average 12.5 weeks for an initial consultation and 23.4 weeks for the first therapy session [1]. McCarthy, McGee and O’Boyle [2] found that most patients (64%) were dissatisfied with these waiting times. Huckert, Hank and Krampen [3] examined changes in psychopathological symptoms from registration for therapy to initial appointment (on average 6 months) and found that the symptoms of the majority of patients (68%) remain unchanged while waiting. However, studies found a significant correlation between waiting time and non-attendance at initial appointment [4, 5]. Hoyer, Helbig and Wittchen [6] found that 41% of patients under current depression treatment had already thought about starting the therapy at an earlier point in their lifetime and showed that long waiting time was one of the three most commonly-mentioned reasons for not even searching for a therapist at all. Thus, the problematic waiting situation increases the rate of late or completely untreated mental disorders, which in turn leads to higher direct and indirect costs [7]. From an ethical, economic and therapeutic perspective, it seems unwise to leave these patients untreated for such a long time.

The provision of low-threshold interventions during waiting time is one option to improve this problem. Psycho-educative information materials [8], bibliotherapy [9, 10], motivational interviewing training [11], group therapy [12] and web-based self-help [13] have already been used as waiting list interventions.

None of these interventions were addressed to patients with physical diseases and comorbid depressive or adjustment disorders. Group interventions seem to be a promising treatment option for the waiting time of these patients, because group-specific factors such as the universality of burden, altruism or interpersonal learning are associated with benefits in coping [14]. To date, there is only one study examining a waiting list group for patients (no special diagnostic indication) to talk about their problems. However, given that this study did not examine effects on mental outcomes and had an attendance rate of only 14% [12], there is a need for a study developing and evaluating a waiting list group intervention for patients with physical diseases and comorbid depressive or adjustment disorders.

The evidence for cognitive behavioral group therapy (CBGT) in depression treatment has been demonstrated in several meta-analyses [15–17], although for patients with physical diseases the body of evidence is less extensive. Many studies in this field have investigated the effects of CBGT on quality of life in all physically ill patients and did not target patients with a mental comorbidity. There is some research on the efficacy of CBGT for depressed outpatients who suffer from the same physical disease. Kelly et al. [18] compared eight sessions CBGT to a waiting list control group in depressed outpatients with HIV and found a medium post-treatment effect (*d* = 0.54^1^) on depression. Evans and Connis [19] compared eight sessions CBGT to a waiting list control group in depressed outpatients with cancer and also found a medium post-treatment effect (*d* = 0.55^1^) on depression. Heckman et al. [20] compared 12 sessions CBGT to a waiting list control group in depressed outpatients with HIV and found a small post-treatment effect (*d* = 0.34), whereas Chesney, Chambers, Taylor, Johnson and Folkman [21] compared 10 sessions CBGT to a waiting list control group in depressed outpatients with HIV and found no significant difference in depression (*d* = 0.16^1^) at post-treatment. To our knowledge, there is only one study investigating CBGT in patients with various physical diseases: Schuster, Rueddel, Keck and Schwarting [22] examined the efficacy of CBGT in inpatients with coronary or orthopedic diseases and comorbid depression, anxiety or somatoform disorders and demonstrated that compared to treatment as usual the group with an additional six sessions CBGT reported significantly lower depression scores at post-treatment (no results provided to calculate effect sizes) and follow-up (strong effect; *d* = 0.89). In summary, existing group interventions for patients with physical diseases and comorbid depression – of which the efficacy has been proven in a randomized controlled trial (RCT) – used either a closed group format with consecutive session contents [18–20] or were designed for an inpatient setting [22]. Therefore, they cannot simply be adopted for an outpatient waiting list group intervention which should be able to continuously include new patients from the waiting list. Our intervention fills this gap.

To overcome waiting times of six months or more for individual therapy in our outpatient clinic for mental disorders in patients with physical diseases, we wanted to establish a waiting list group intervention as a first step of treatment. We developed STEpS, a half-open eight-session CBGT for patients with various physical diseases and comorbid depressive or adjustment disorders waiting for individual therapy [23] and conducted a RCT to examine its efficacy. The primary outcome of the study was depression; secondary outcomes were global psychological distress and health-related quality of life. We hypothesized that the STEpS group would be superior in these outcomes compared to a waiting list control group (CG) at the end of treatment as well as at 2-month follow-up, although the benefits would probably be smaller.

## Methods

We conducted a two-armed RCT to compare a waiting list group intervention (STEpS) with a waiting list control group (CG). The study took place at the outpatient clinic of the Institute of Psychology at the Albert-Ludwigs-University of Freiburg, which is specialized in the treatment of mental disorders in patients with chronic physical diseases. Measurement points were taken at baseline (before initial consultation, T_1_), post-treatment (T_2_) and 2-month follow-up (T_3_). The study protocol was approved by the ethics committee of the Albert-Ludwigs-University of Freiburg [23].

### Participants

The target group of this study was patients with self-reported chronic physical diseases such as cardiovascular diseases, stroke, cancer, chronic respiratory diseases or diabetes and comorbid depressive disorders or adjustment disorders of subtype depressed mood or subtype mixed anxiety and depressed mood. Adjustment disorders were included due to their high prevalence in patients with chronic physical diseases [24, 25]. Adjustment disorders of the subtypes depressed mood or mixed anxiety and depressed mood can be seen as subthreshold depression [26] where the symptoms are attributable to a stressful event differing from depressive disorders in symptom severity but not necessarily in duration because in case of chronic physical diseases the stressful event is persistent.

### Recruitment and assessment of eligibility

We recruited study participants from patients who contacted our outpatient clinic for individual therapy. As we did not have enough registrations at our clinic to fill a therapy group when randomizing patients with an allocation ratio of 1:1 to intervention or control group, we had to recruit additional study participants. Therefore, we additionally recruited participants through newspaper announcements. Eligible participants were a) 18 years and older, b) reported a chronic physical disease, c) met the criteria for adjustment disorder with depressed mood or adjustment disorder with mixed anxiety and depressed mood, depressive episode (mild to severe, single or recurrent) or dysthymia, all according to the Diagnostic and Statistical Manual of Mental Disorders IV text revision (DSM-IV-TR) criteria as measured with Structured Clinical Interviews for DSM-IV Axis I Disorders (SCID-I; [27] and d) had sufficient skills to participate in a group setting (e.g. the ability to adhere to group rules), which was evaluated by clinical assessment in the initial consultation. Patients with acute suicidal ideation, bipolar affective disorder, cyclothymic disorder, comorbid substance abuse, schizophrenia, dementia, learning disability, ongoing psychotherapy or insufficient knowledge of German were excluded from the study. All participants were initially screened through a telephone call, an initial consultation and a SCID-I interview (for more details, see Ruesch et al. [23]. There was no cost and no compensation for study participation. All study participants gave written informed consent.

### Randomization

Block randomization strives for similar group sizes throughout the trial. In order to guarantee a continuous group size in the STEpS group, we used block randomization with a block size of 20 and an allocation ratio of 1:1. The allocation sequence was created using computer-generated random numbers by an independent researcher. We used a sealed envelope system to guarantee that the staff involved in enrollment and allocation did not know the allocation sequence.

### STEpS

Study participants allocated to STEpS participated in eight sessions CBGT while waiting for individual therapy. Before joining the group session for the first time, patients had a short consultation with the group therapist. This served to inform them about the procedures of the group, the group rules and reduce any fears. To ensure an immediate entry to the intervention, the group was defined as half-open, which meant that new group members could enter with every new module (every second session). In order to enhance social exchange, the group size was limited to eight patients. All groups were led by one psychotherapist licensed in CBT and very experienced in leading groups. Potential deviations from protocol were recorded in writing.

The group intervention comprised eight weekly sessions, each of 100 min. The length of the intervention was determined by content-related reasons. The intervention covered four different modules: 1. behavioral activation, 2. cognitive restructuring, 3. coping with illness and social support, 4. self-esteem and meaning. Each module comprised two sessions (for a detailed session overview, see Table 1). The module topics and interventions were evidence-based and tested in a pilot group. After follow-up, patients were offered individual therapy at our clinic.

**Table 1:** Overview of STEpS (compare Ruesch et al. [23])

Session	Topic	Content
1	Behavioral activation I	- Psychoeducation about the relationship between behavior and emotion: Explanation of the downward mood spiral [37] - Selection of individual positive activities from a checklist [38] and rating of the selected activities - Scheduling three positive activities for the next week
2	Behavioral activation II	- Review of homework - Information about the effects of physical activity on depression, physical health, and well-being - Brainstorming on personal health objectives and possible physical activities to reach these objectives - Introduction of the MoVo concept [39] and illustration of the different steps in the motivational and volitional process of physical activity. Personal elaboration of the different steps (e.g. building a concrete intention, generating implementation intention, developing counter strategies for anticipated barriers)
3	Cognitive restructuring I	- Psychoeducation about the relationship between thoughts and depressive mood/emotion - Introduction of the ABC model [40] by means of a role-play - Practicing the categorization in A (activating event), B (beliefs), and C (consequences) using patients’ examples. Further explanation and illustration of the relevance and changeability of cognitions
4	Cognitive restructuring II	- Introduction of typical cognitive distortions/irrational beliefs [41] - Identification of individual maladaptive thoughts and development of alternative thoughts
5	Coping with illness	- Information about the coping concept. Psychoeducation about the person and situation specificity of adaptive coping - Collection of disease-related stressors (e.g. pain, disabilities, unclear illness course). Small group discussion about helpful coping strategies concerning one stressor. Gathering the coping strategies for the different stressors - Group conversation about personal intentions to try other strategies
6	Social Support	- Introduction to the concept of social support and its relevance concerning chronic illness - Reflection on the personal social relations through drawing a social atom [42] and clarification about who in the network gives social support and how - Group conversation about the social network and received social support (e.g. wishes of change)
7	Self-esteem	- Joint development of a definition of self-esteem - Introduction of the four pillar model of self-esteem (self-acceptance, self-confidence, social skills, social networks) [43] - Reflection of personal strengths and weaknesses in different aspects of the self (e.g. me as wife, me as professional) - Wheel of life exercise to reflect the actual and target distribution of life energy on different life domains
8	Meaning	- Introduction of the meaning concept - Reflection and group conversation about meaningfully moments in life - Illustration that everyday activities (e.g. the job) can be done technically or meaningful and that meaning can be a resource for coping - Reflection and group conversation about ideas for more sense of meaning in life

### Control group (CG)

Study participants allocated to the CG passed the normal waiting process of our outpatient clinic and had no intervention while waiting for individual therapy. After follow-up, patients were offered individual therapy at our clinic. If the CG patients were interested, they could receive STEpS after follow-up.

### Measures

The primary outcome was self-reported depression, while secondary outcomes were self-reported global psychological distress and health-related quality of life.

*Depression* was measured by the depression subscale of the German version of the Hospital Anxiety and Depression Scale (HADS-D; [28]). This scale was used because it was developed especially for the target group of patients with comorbid physical diseases. Depression sum-scores ranging from 0 to 21 were computed, whereby higher sum-scores indicated higher depression. Cronbach’s α was.78, indicating acceptable internal consistency.

*Global psychological distress* was assessed by the German version of the Brief Symptom Inventory (BSI; [29]). The inventory comprised 53 items with symptoms from nine domains. Respondents were asked to rate their symptoms within the last seven days on a five-point scale from 0 (“not at all”) to 4 (“extremely”). The Global Severity Index (GSI) indicating global psychological distress was computed by calculating the mean of all items. Cronbach’s α for the GSI was.95, indicating excellent internal consistency. In addition, we measured the global distress level using the visual analogue scale of the German version of the NCCN Distress Thermometer (DT; [30]). Respondents had to indicate their current distress level within the last week on an -point Likert scale in a visual thermometer ranging from 0 (no distress) to 10 (extreme distress).

*Health-related quality of life* was assessed by the German version of the Short Form Health Survey (SF-12; [31]). Physical and Mental Composite Scores ranging from 0 to 100 were computed, whereby higher scores indicated higher subjective physical or mental health. Cronbach’s α for the SF-12 was.76, indicating an acceptable internal consistency.

### Sample size calculation

A sample size of 128 participants is needed to detect a main group effect of *f* = 0.25 when adjusting for baseline severity and assuming a power (1-β) of 0.8 and an alpha of 0.05. Sample size calculation was conducted by G*Power 3 software.

### Statistical analysis

According to the Consolidated Standards of Reporting Trials (CONSORT), all analyses were based on the intention-to-treat (ITT) sample of 76 randomized patients. Missing data was handled by multiple imputation [32, 33]. Missing values in the clinical outcomes were imputed using a Markov Chain Monte Carlo algorithm with 20 estimations per missing value based on demographics, clinical characteristics and valid outcomes of all measurement points. The 20 analyses were pooled for a single overall estimate of the results.

Demographic and clinical characteristics of the study sample were analyzed using χ^2^-tests or Fisher’s exact test for nominal variables and unpaired t-tests for continuous variables. Differences in the outcomes between STEpS and CG at post-treatment (T_2_) and follow-up (T_3_) were analyzed using between-group analyses of covariance (ANCOVA) with baseline scores of the outcome variables as covariates. As SPSS 20 is unable to pool *F*-tests, we reformulated the ANCOVA as a regression model. Alpha was set at.05 for all analyses. Cohen’s *d* was calculated by dividing the difference between the estimated group means by the pooled standard deviation of the observed scores. According to Cohen [34], *d* = 0.2 indicated a small effect, *d* = 0.5 a medium and *d* = 0.8 a large effect. To determine the influence of missing data on the results of the ITT analysis, we additionally performed available case analyses including all available data in each analysis (pairwise deletion of missing values). All analyses were conducted with IBM SPSS Statistics 20.

## Results

### Patient flow

**Fig. 1:**
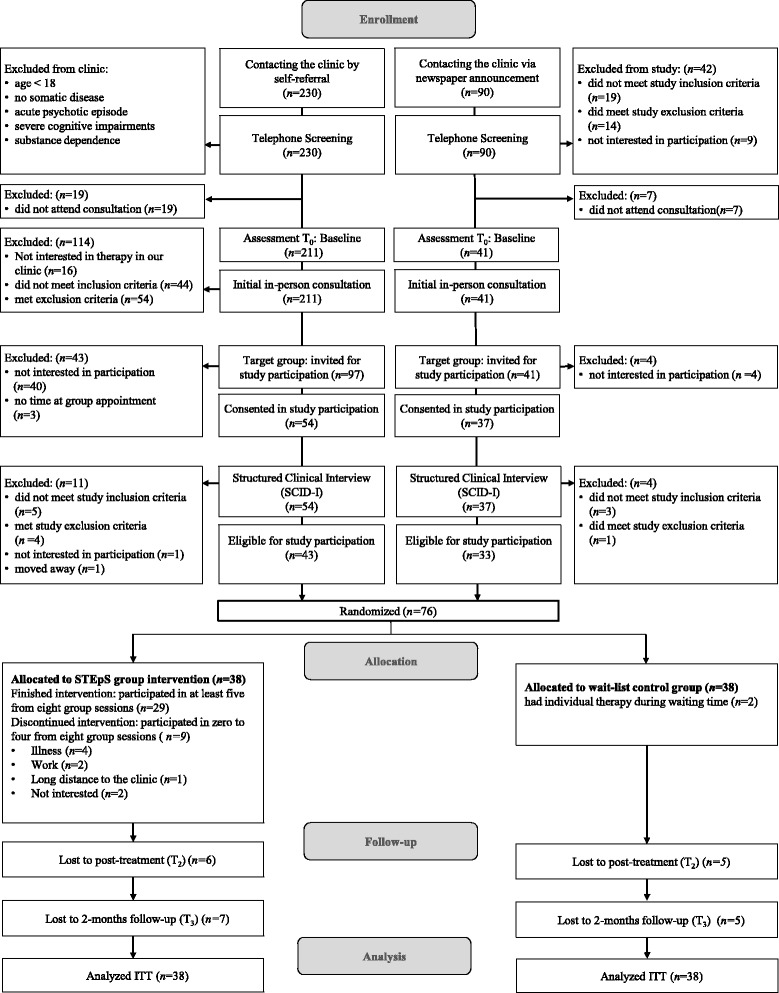
Study flow diagram

We had two different admission pathways (see Fig. 1). In the recruitment period (August 2013 to December 2014), a total of 230 patients registered at our clinic to start psychotherapy (self-referral subgroup) and 211 of them attended an initial consultation. Sixteen of them did not want to be on the waiting list for therapy. An additional 98 patients were excluded from the study because they did not fulfill inclusion criteria or met one of the exclusion criteria. Of the 97 remaining patients, 57 were interested in participating in the study, three of whom had time constraints at the group appointment. Thus, 54 patients consented to participate in the study and were screened with the SCID-I. After the SCID-I, another 11 patients had to be excluded. Eventually, 43 of these patients fulfilled eligibility.

Ninety patients contacted the clinic due to the newspaper announcement (newspaper subgroup). We excluded 42 patients in the telephone screening based on eligibility criteria and seven patients did not attend the initial consultation. Of the 41 patients who attended the initial consultation, 37 consented to study participation. After the SCID-I, another four patients had to be excluded. Ultimately, 33 of these patients fulfilled eligibility. Thus, 76 patients were included in the study and randomized to the treatment conditions. A detailed overview of the patient flow is given in Fig. 1.

Twenty-nine out of 38 patients (76%) in the STEpS group completed the intervention and participated in at least five out of eight group sessions. Nine patients (24%) did not complete the intervention and attended zero to four sessions. Seven patients mentioned external reasons for non-completion (illness (*n* = 4), no time due to job (*n* = 2), long drive to the clinic (*n* = 1)) and two patients mentioned no interest in further participation (*n* = 2). Sixty-five percent of the self-referral subgroup (13 out of 20 patients) completed the intervention and 89% of the newspaper subgroup (16 out of 18 patients) completed the intervention. Completion rates did not significantly differ between the two subgroups (Fisher’s exact test, *n* = 38, *p* = .088).

Dropout rates at post-treatment and follow-up were relatively low: 11 patients (14%) were lost to T_2_ and 12 patients (16%) were lost to T_3_ (see Fig. 1)_._ Seven patients only completed T_1_, four patients completed T_1_ and T_3_, and five patients completed T_1_ and T_2_ and dropped out at T_3._ Dropout rates did not significantly differ between the self-referral subgroup and the newspaper subgroup (post-treatment: 19% vs. 9%, (χ^2^(1, *n* = 76) = 1.37, *p* = .243); follow-up: 19% vs. 12%, (χ^2^(1, *n* = 76) = 0.59, *p* = .442)).

### Baseline characteristics

The demographic and clinical characteristics of the study sample at baseline are presented in Table 2. The mean age of the study sample was 54.5 years, with a range from 18 to 77. The majority of participants (70%) were female. More than half the patients (59%) were married or in a relationship. On average, they spent 10.6 years in school. About half the patients (49%) were employed either full or part time or were in training, 29% were retired and 17% were on sick leave. All patients reported a chronic physical disease, most commonly diseases of the musculoskeletal system, neoplasms, diseases of the nervous system and diseases of the circulatory system. Thirty-six per cent of the sample reported more than one chronic physical disease. The majority of the patients (71%) suffered from a depressive episode (single or recurrent): 20% had a mild, 29% a moderate, 3% a severe and 20% a partial remitted depressive episode. Seventeen percent of the patients had an adjustment disorder, 9% a dysthymic disorder and 3% other diagnosis (mixed anxiety and depression or recurrent brief depression).

**Table 2:** Characteristics of the study sample at baseline (*N* = 76)

		STEpS	CG^a^	Total	*t*/χ2	*p*
		(*n* = 38)	(*n* = 38)	(*N* = 76)		
Gender	Male	10 (26.3%)	13 (34.2%)	23 (30.3%)	χ2(1) = .561	.454
	Female	28 (73.7%)	25 (65.8%)	53 (69.7%)		
Age	*Mean* in years *(standard deviation)*	55.4 (14.1)	53.6 (12.3)	54.5 (13.2)	*t*(74) = .615	.540
Family status	Single	5 (13.2%)	7 (18.4%)	12 (15.8%)	χ2(1) = 1.363	.506
	Divorced, separated or widowed	8 (21.1%)	11 (28.9%)	19 (25.0%)		
	Married or in relationship	25 (65.8%)	20 (52.6%)	45 (59.2%)		
Duration of school education	*Mean* in years (*standard deviation)*	10.7 (1.4)	10.6 (1.7)	10.6 (1.6)	*t*(70) = .095	.925
	Did not finish school	2 (5.3%)	1 (2.6%)	3 (3.9%)		
	Still in school	1 (2.6%)	0 (0%)	1 (1.3%)		
Employment status	Employed full time	7 (18.4%)	9 (23.7%)	16 (21.1%)	Fisher’s exact test	.994
	Employed part time	10 (26.3%)	9 (23.7%)	19 (25.0%)		
	In training	1 (2.6%)	1 (2.6%)	2 (2.6%)		
	Unemployed	1 (2.6%)	1 (2.6%)	2 (2.6%)		
	On sick leave	7 (18.4%)	6 (15.8%)	13 (17.1%)		
	Retired	11 (28.9%)	11 (28.9%)	22 (28.9%)		
	Housekeeping	1 (2.6%)	1 (2.6%)	2 (2.6%)		
Physical diagnosis^b^ *(multiple answers possible)*	Musculoskeletal system and connective tissue	16 (42.1%)	17 (44.7%)	33 (43.4%)	Fisher’s exact test	.129
	Neoplasms	11 (28.9%)	8 (21.1%)	19 (25.0%)		
	Nervous system	11 (28.9%)	7 (18.4%)	18 (23.7%)		
	Circulatory system	7 (18.4%)	7 (18.4%)	14 (18.4%)		
	Endocrine, nutritional and metabolic diseases	0 (0%)	8 (21.1%)	8 (10.5%)		
	Skin and subcutaneous tissue	3 (7.9%)	2 (5.3%)	5 (6.6%)		
	Digestive system	3 (7.9%)	1 (2.6%)	4 (5.3%)		
	Respiratory system	2 (5.3%)	1 (2.6%)	3 (3.9%)		
	Eye and adnexa	2 (5.3%)	1 (2.6%)	3 (3.9%)		
	Genitourinary system	1 (2.6%)	2 (5.3%)	3 (3.9%)		
	Injuries, poisoning and external causes	0 (0%)	2 (5.3%)	2 (2.6%)		
	More than one diagnosis	13 (34.2%)	14 (36.8%)	27 (35.5%)		
Diagnosis (SCID-I)^c^	Depressive episode	27 (71.1%)	27 (71.1%)	54 (71.1%)	Fisher’s exact test	.195
	*Mild*	*11 (28.9%)*	*4 (10.5%)*	*15 (19.7%)*		
	*Moderate*	*10 (26.3%)*	*12 (31.6%)*	*22 (28.9%)*		
	*Severe*	*0 (0%)*	*2 (5.3%)*	*2 (2.6%)*		
	*In partial remission*	*6 (15.8%)*	*9 (23.7%)*	*15 (19.7%)*		
	Adjustment disorder with depressed mood or with mixed anxiety and depressed mood	5 (13.2%)	8 (21.1%)	13 (17.1%)		
	Dysthymic disorder	4 (10.5%)	3 (7.9%)	7 (9.2%)		
	Others	2 (5.3%)	0 (0%)	2 (2.6%)		
	More than one diagnosis	14 (36.8%)	19 (50.0%)	33 (43.4%)		
Previous treatment	Under Medication^d^	15 (39.5%)	15 (39.5%)	30 (39.5%)	Fisher’s exact test	.450
	Previous psychotherapy^e^	22 (57.9%)	19 (50.0%)	41 (53.9%)	Fisher’s exact test	.349
Admission to clinic	Newspaper announcement	18 (47.4%)	17 (44.7%)	35 (46.1%)	χ2(1) = .053	.818
	Self-referral	20 (52.6%)	21 (55.3%)	41 (53.9%)		

Notes:

^a^CG = control group

^b^categories according to International Classification of Diseases (ICD-10)

^c^SCID-I = Structured Clinical Interview for DSM-IV

^d^Medication of *n* = 5 patients is unknown

^e^Previous psychotherapy of *n* = 4 patients is unknown

Nearly half the patients (43%) had at least one more axis I disorder, most often anxiety disorders or somatoform pain disorders. Forty per cent of the patients were under ongoing psychopharmaceutic medication and more than half the patients (54%) had received previous psychotherapy. Forty-four per cent of those with previous psychotherapy indicated that they had it within the last 2 years. There were no significant differences between the STEpS group and CG in demographic and clinical variables (see Table 2).

### Changes in primary and secondary outcomes (ITT analysis)

**Fig. 2:**
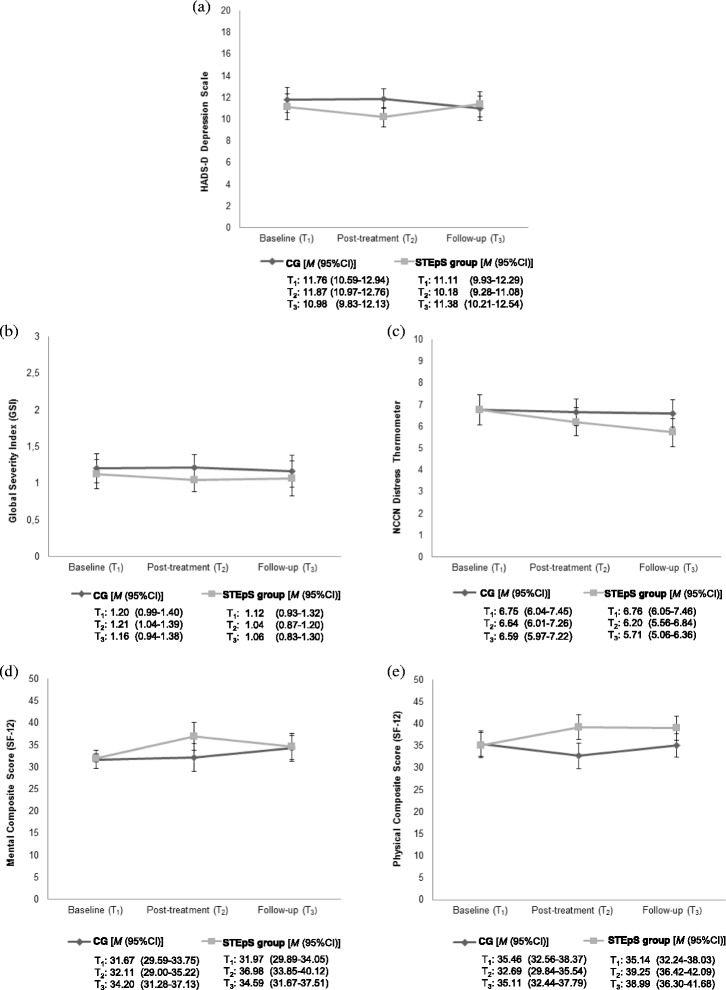
Estimated means with the 95% confidence interval of (**a**) depression, (**b**) global psychological distress (GSI), (**c**) global distress (DT) (**d**) mental health and (**e**) physical health in the ITT sample (*N* = 76). Values at T_2_ and T_3_ are adjusted for baseline scores. Abbreviations: CG = control group; *M* = mean; CI = confidence interval

The estimated mean values and the 95% confidence interval of the primary and secondary outcome variables at baseline, post-treatment (adjusted for baseline) and follow-up (adjusted for baseline) are presented in Fig. 2.

Figure 2a shows the changes in the primary outcome. The STEpS group reported significantly lower depression scores compared to the CG at post-treatment (*B* = −1.69, *SE =* 0*.*65, CI = −2.96 – -0.42, *T* = −2.60, *p* = .009). The effect size was small (*d =* 0.37). At follow-up, the groups did not significantly differ in depression (*B* = 0.40, *SE* = 0.85, CI = −1.26 – 2.06, *T* = 0.47, *p* = .637).

Figure 2b-f refers to the secondary outcomes. Figure 2b shows the changes in global psychological distress measured with the GSI. The STEpS group and the CG did not significantly differ in GSI at post-treatment (*B* = −0.18, *SE =* 0.12, CI = −0.42 – 0.07, *T* = −1.43, *p* = .153) and follow-up (*B* = −0.10, *SE =* 0.17*,* CI = −0.44 – 0.25, *T* = −0.56, *p* = .577). Figure 2c shows the changes in global distress measured with the visual analogue scale of the DT. The STEpS group and the CG did not significantly differ in global distress at post-treatment (*B* = −0.44, *SE =* 0.46, CI = −1.33 – 0.46, *T* = −0.95, *p* = .340) and follow-up (*B* = −0.88, *SE =* 0.45*,* CI = −1.77 – 0.01, *T* = −1.95, *p* = .051).

Figure 2d shows the changes in mental health. The STEpS group reported significantly higher mental health scores compared to the CG at post-treatment (*B* = 4.87, *SE =* 2.25*,* CI = 0.46 – 9.28, *T* = 2.16, *p* = .030). The effect size was small (*d* = 0.47). At follow-up, the groups did not significantly differ in mental health (*B* = 0.39, *SE =* 2.11*,* CI = −3.75 – 4.53, *T* = 0.18, *p* = .854). Figure 2e shows the changes in physical health. The STEpS group reported significantly higher physical health scores compared to the CG at post-treatment (*B* = 6.56, *SE =* 2.05*,* CI = 2.56 – 10.57, *T* = 3.21, *p* = .001) and follow-up (*B* = 3.88, *SE =* 1.94*,* CI = 0.07 – 7.68, *T* = 1.10, *p* = .046). Effect sizes were medium (*d* = 0.70) at post-treatment and small (*d* = 0.43) at follow-up.

### Available cases analysis

Available cases analysis revealed similar results for the primary outcome: We also found a small effect on depression at post-treatment (*n* = 64, *B* = −1.70, *SE =* 0*.*64, CI = −2.97 – -0.43, *T* = −2.68, *p* = .009, *d* = 0.37) and no effect on depression at follow-up (*n* = 63, *B* = 0.74, *SE =* 0.83*,* CI = −0.92 – 2.39, *T* = 0.89, *p* = .376). The results for secondary outcomes did not deviate from the results of the ITT analysis: the *B* values (= differences between the two adjusted means) for all secondary outcomes and all time points were within the 95% confidence interval of the *B* values of the ITT analysis.

## Discussion

The aim of this RCT was to evaluate the efficacy of STEpS, an eight-session CBGT for outpatients with various physical diseases and comorbid depressive or adjustment disorders on a waiting list for individual therapy. As hypothesized, patients receiving STEpS showed significantly lower depression scores at post-treatment compared to the CG. The effect size achieved was small (*d* = 0.37). As there is no other study examining CBGT in a comparable sample of depressed outpatients with various physical diseases, we have to compare our findings with the results of studies that examined CBGT either in depressed outpatients who suffered from the same physical disease (HIV or cancer) or in inpatients with various physical diseases and mental comorbidity. Studies on the efficacy of CBGT in depressed outpatients who suffered from the same physical disease have revealed heterogeneous effects on depression (*d* = 0.16 [21], *d* = 0.34 [20], *d* = 0.54 [18], *d* = 0.55 [19]). Different effect sizes across these studies may be partly influenced by different strategies of handling missing data (using a completer analysis [18, 19] vs. imputing missing values by last observation carried forward [20]). Our findings strengthen the position that CBGT is efficacious in reducing depression in these patients. Compared to Schuster et al. [22] – who compared an additional six sessions CBGT to treatment as usual in inpatients with different physical diseases and mental comorbidity and revealed a strong effect on depression at 6-month follow-up – our effect on depression was considerably smaller. However, their strong effect size should probably be interpreted with caution, for two reasons: first, their inpatient CBGT explicitly focused on encouraging patients to start subsequent individual outpatient psychotherapies and thus it seems very likely that the follow-up effect also reflects improvements through these subsequent therapies; and second, the results were based on a completer sample with unknown size and thus might overestimate the effect. Comparisons with studies on other waiting list interventions cannot be drawn because these studies did not report effects on mental outcomes [7].

Effects on secondary outcomes were mixed. STEpS resulted in higher health-related quality of life scores at post-treatment. The effect on self-reported mental health was small (*d* = 0.47) and the effect on self-reported physical health was medium (*d* = 0.70). However, STEpS had no effect on global psychological distress. This may be explained by the severe burden due to the multimorbidity of the sample. Patients reported at least a depressive or an adjustment disorder, although more than half had further mental diagnoses. Taking this into account, it is unsurprising that our intervention – which mainly focused on dealing with depressive symptoms – did not lead to reduced global psychological distress.

The benefits of the STEpS group in depression and mental health did not remain stable. Eight weeks after the end of the treatment, there were no significant differences between the groups. This is in line with findings from the above-mentioned studies examining CBGT in patients who suffered from the same physical diagnosis, which also failed to reveal long-term effects (at 3-month, 4-month or 6-month follow-up) on depression [18–20]. One reason for the symptom deterioration could be that the support of the group terminated and the individual therapy did not start right away, whereby patients may have felt left alone again. Receiving something that seems helpful and then having it taken away may have triggered this adverse effect. Indeed, when considering the multimorbidity of the sample, it again becomes quite understandable why eight sessions CBGT did not lead to follow-up effects. To prevent this rebound in depression and mental health after group termination, it could be promising to reduce the interval between the last group session and the beginning of the individual therapy by a) prolonging the group therapy to about 12 sessions, b) adding booster sessions or c) increasing the intervals between the eight sessions. Future research should clarify whether this could lead to better follow-up effects. It seems a little counterintuitive that STEpS had the largest and most stable effect on self-reported physical health. One hypothesis for this is that the exchange with other patients suffering from different physical diseases and seeing one’s own health in relation to the poor health of others could have resulted in a re-evaluation of one’s own health status. Another hypothesis could be that the behavioral activation intervention led to new positive experiences that changed the evaluation of their health status.

About half the patients in our sample had previous psychotherapy. Further analyses showed that IG patients with or without previous treatment experience did not significantly differ in depression at post-treatment (*B* = −1.61, *SE =* 0.73, CI = −1.59 – 1.27, *T* = −0.22, *p* = .825). Thus, the group intervention even seemed to be helpful for patients with previous treatment experience.

Overall, a large spectrum of depressive disorders is represented in our study sample. Only severe depressive episodes were underrepresented, although this in turn is representative for the German outpatient setting [35]. Moreover, the study sample had a large spectrum of physical diseases and mental comorbidities and were allowed to have previous psychotherapy experience or medication. All of these points enhance the external validity of our study and contribute to the comparability of the study sample to the routine patient care [36].

The fact that 57 of 97 (59%) of the self-referred patients were interested in participating in the study may indicate the strong need for waiting list interventions. Compared to the 14% who made use of the waiting list group in the study of Stone and Klein [12], this rate emphasizes the attractiveness of our intervention. The STEpS group sessions were highly accepted. This can be concluded from the attendance rate of 76% and the aforementioned predominantly external reasons for non-completion. The rate of patients who attended seven or eight out of eight sessions (58%) also supports acceptance. The attendance rate of the subgroup of self-referred patients was lower (65%), which might indicate a lower attendance rate under routine conditions. It must also be noted that 42% of the target group refused study participation. One of the main reasons for refusal seemed to be the group setting. Thus, it could be hypothesized that the implementation of group therapy as waiting list intervention in standard routine may not serve the needs of all patients.

### Implementation in routine care

The STEpS waiting list intervention could easily be transferred to routine outpatient care, although it must be considered that a waiting list intervention for patients with chronic physical diseases and comorbid mental disorders would only hold interest and be feasible for clinics or psychotherapist specialized in this field. The costs for the STEpS group sessions were covered by health insurances. A recent reform of the German health care system aimed to strengthen group therapy and facilitates the implementation of STEpS in routine care: fees for group sessions were increased by 20% and it is no longer a problem to treat patients only by group therapy or provide group and individual therapy by different therapists. Even if STEpS could only be implemented at the cost of 2-3 individual sessions per week, its implementation might be worthwhile because more patients can be treated within the given resources and therapy can be provided sooner.

### Limitations

This study has several limitations. First, due to limited resources we did not achieve the a priori estimated sample size of 128 patients and thus the study may be underpowered: the observed effects are still valid, although it remains uncertain whether we detected all effects. Therefore, we based all analyses on the ITT sample of 76 patients and used multiple imputations to handle missing data. Second, we deviated from the protocol and did not adjust alpha for multiple testing. Taking into account the loss of power related to the lower sample size, alpha adjustment was not feasible. Therefore, the risk of an alpha error must be considered when interpreting the results. Third, the results could be biased due to the recruitment methods in this study. To eliminate the risk that differences in symptom severity or motivation could be possible, we analyzed the data with t-tests and found no baseline differences in the outcomes between patients recruited from our clinic and those recruited from the newspaper announcement. We have already demonstrated that patients did not significantly differ in dropout or intervention completion according to their recruitment to the study. Further analyses also showed they did not have differential intervention benefits. Fourth, therapist adherence was not assessed by an external rating. Although there were no documented deviations from the manual, unconscious deviations due to a learning process might be possible. Fifth, we did not assess treatment for physical health problems. Differences between IG and CG in the treatment of physical health may have also influenced the differences in depression and quality of life, although as randomization in the assessed domains worked well this bias does not seem very likely. Sixth, the design of the study did not enable examining whether the STEpS intervention itself or social support while waiting led to the improvements in depression and health-related quality of life. Further investigations should compare STEpS to a non-specific social support group to answer this question.

Furthermore, there are several limitations of the STEpS intervention. First, the intervention focused only on depression and adjustment disorder and is unable to address further mental comorbidities. Second, the group setting limits the possibility to respond to the individual needs of the patients. Third, a waiting list group intervention needs an open or half-open setting, although this could be to the detriment of group cohesion. Fourth, a group intervention with more sessions (e.g. 12 sessions) would shorten the interval between the last group session and the beginning of individual therapy and might lead to better results.

## Conclusions

This study contributes to the limited evidence on the efficacy of CBGT in patients with physical diseases and comorbid depressive or adjustment disorders. It is the first RCT to examine the efficacy of a waiting list group intervention, as well as the first RCT to examine the efficacy of CBGT in outpatients with various physical diseases and comorbid depressive or adjustment disorders. The waiting list group intervention proved effective in improving depression and health-related quality of life in the short term, but not at 2-month follow-up. Even though the intervention revealed only small to medium short-term effects on these outcomes, its implementation as a waiting list intervention could help multimorbid patients to handle long waiting periods for individual therapy. Therefore, the presented waiting list intervention contributes to a main problem in outpatient mental care in Germany. Future research should focus on methods to obtain better follow-up effects.

## Abbreviations

ANCOVA: Analyses of covariance
BSI: Brief Symptom Inventory
CBGT: Cognitive behavioral group therapy
CG: Control group
CI: Confidence interval
CONSORT: Consolidated standards of reporting trials
DSM-IV-TR: Diagnostic and Statistical Manual of Mental Disorders, fourth edition, text revision
DT: NCCN Distress Thermometer
GSI: Global Severity Index
HADS-D: Hospital Anxiety and Depression Scale
ITT: Intention-to-treat
RCT: Randomized controlled trial
SCID-I: Structured Clinical Interview for DSM-IV Axis I Disorders
SF-12: Short Form Health Survey

## Footnotes

1 Cohen’s *d* was calculated from the presented results.
